# Medical Education Journal Club: Two years’ experience at King Saud University

**DOI:** 10.12669/pjms.335.13221

**Published:** 2017

**Authors:** Samina A. Khan, Mona M. Soliman

**Affiliations:** 1 Samina A. Khan, M.Sc. Medical Education Department, College of Medicine, King Saud University, Riyadh, Saudi Arabia; 2 Mona M. Soliman, MBBS, MSc, PhD. Chairman, Medical Education Department Professor of Physiology and Medical Education, Consultant Cardiac Physiology, Medical Education Department, College of Medicine, King Saud University, Riyadh, Saudi Arabia

**Keywords:** Medical education, Journal club, Evidence based medical education

## Abstract

**Objective:**

Medical Education Journal Club is an evidence-based approach to teach and learn critical appraisal techniques on available literature. This study evaluates the implementation and experience of two academic years of Journal club at Medical Education Department, King Saud University.

**Methods:**

We started JC in 2015 at medical education department, KSU. An invitation with a published paper and event poster were sent 2 weeks prior of the session to participants. A traditional one-group posttest design with open item survey were conducted at the end of every session.

**Results:**

A total of 12 sessions were conducted in total. The average attendance of 26 (Male: 42/79, 53.1%) and (Female: 31/79, 39.2%) with mix of professors, associate and assistant professors. The MEJC had a positive effect on participant’s session expectations (45/79, 92.4%), and had increased their knowledge of the field (73/79, 92.4%). It was observed that the attendance of event depends on the speaker for the event. The sessions have also arisen the need of trainings and other scientific activities.

**Conclusions:**

MEJC is an educational activity that can play important aspect in providing high quality healthcare teachings. We conclude that the success and consistency of MEJC depends on speaker. It commensurate the audience interest to attend and learn. While proper advertisement of event, and regular attendance also plays a vital role in this regard.

## INTRODUCTION

Journal Clubs (JC), better defined as conventional method of discussion and evaluation of scientific literature in academic training curriculum^1^,^2^. It has also been a method of continuing medical education for over hundred years..^3^ Study details Sir William Osler was the founder of first Journal Club called North American JC at McGill University (Montreal, Canada) back in 1875.^3^,^4^ Journal Club plays an important role in changing the culture of medical education,^5^ concepts of new learning and teaching methods^6^ and clinical practice proficiency.7 Studies suggests clinician’s involvement in JC as an important part of their research design, implementation, and dissemination to add a value to the literature of evidence-based care.^8^ It also plays a crucial role in exposure to critical appraisal techniques and medical statistics in literature.^9^,^10^ The aim of the study was to evaluate the implementation and experience of two years of 1^st^ journal club in Medical Education, College of Medicine, King Saud University.

## METHODS

### Setting, origin and development of MEJC

In February 2015, we started first JC focused on medical education, specifically for the Medical Education Department, in College of Medicine of King Saud University (KSU). With wide range of colleges, and advance research centers, KSU is one of the top tertiary institution of Saudi Arabia. Medical Education Department, in College of Medicine of KSU has different units of Medical Informatics, E-Learning, Simulation, Problem Based Learning, curriculum development, and Undergraduate Elective faculty development unit. The goals of the MEJC were: to critically evaluate; discuss how scientific literature and research can be translated into practice; education; administration; and research in different area of Medicine.

### MEJC Methodology

The MEJC is a monthly one-hour activity, in a regular day and time. Started in February 2015 till March 2017 and completed a total of 12 sessions.

### Formal Invitation

A well planned “Presenter’s Guidelines” was designed to send, forth with formal invitation email, to the presenting unit of the department by MEJC coordinator, which details the rules of time management, presentation outline, and article/topic selection instructions. Along with this guideline manual, an additional information sheet was provided to summarize the meeting details, 4 weeks before the session. Invitations were also circulated to different experts in the Medical Education and the medical college region. National and International speakers outside of Medical Education department were also welcomed time to time to share their knowledge.

### Advertisement and the event

The MEJC coordinator, 2 weeks’ prior to the meeting sends a formal invitation, including the advertising poster, and related documents to be discussed to all the staff of medical education.

A short questionnaire based on traditional one-group posttest design using a self-administered structured questionnaire in the English language was designed to evaluate this academic activity. The reason to use this design is that it takes less time and we were evaluating first JC in Medical Education in KSU. Questionnaire were sent exactly an hour after the end of the session via email. The questionnaire also included open ended questions related to suggestions about the MEJC and the topic covered. Thematic analysis was performed to evaluate these open ended questions which will be used to shape the future events.

### Ethical aspects

Selection and approval of article/topic of presentation were performed with proper protocol. Written informed consent was obtained. The data were collected, managed anonymously and analyzed in the aggregate.

## RESULTS

### Response Rate and Demographic Information

A total of twelve sessions took place in time of two academic years. Average attendance was 26, consisting (Male: 87/156, 55.7%) and (Female: 69/156, 44.2%). Of the 156 participants we approached to participate in the study at end of every session, 79 (50.6%) completed the survey, whereas 77 of 156 (49.3%) participants decided not to take part due to their own personal reasons.

Of these participants, males were 42 (53.1%) and Females were 31(39.2%) in number which consisted of Assistant professors (09/79, 11.39%), Associate professors (04/79, 5.06%), Clinical instructors (07/79, 8.86%), Professors (14/79, 17.72%), researchers (09/79, 11.39%) and 06/79 (7.59%) of other different positions.Table-1

**Table-I:** Demographic data of MEJC participants that participated in the survey.

*Gender:*	*Male: 42/79 (53.1%) and Female: 31/79 (39.2%)*	
Academic Positions:	*N* = 49	Professors = 14 (28.5%), Associate professors = 04 (8.1%), Assistant professors = 09 (18.3%)
Professional Field:	*N* = 47	Medicine = 43 (91.4%), Pedagogy = 26 (48.9%)
Medical Specialty:	*N* = 47	Physiology = 6 (12.7%), Radiology = 3 (3.6%), Family and Community = 3 (3.6%), OB/Gyne = 3 (3.6%), Surgery = 3 (3.6%), Cardiac science = 1 (2.1%), E.N.T = 1 (2.1%), Pathology = 1 (2.1%)

*N= Number of respondents

### Sessions evaluation

A good number of participants (45/79, 92.4%) agreed that the MEJC sessions met their expectations, where (78/79, 98.7%) strongly agreed that the content presented were helpful, and added knowledge to their field (73/79, 92.4%). In like manner, (75/79, 94.9%) strongly agreed about the sessions to be well organized, and were of optimum length (71/79, 89.8%).

Participants were also encouraged to comment, question or show any concern regarding the session they attended. Thematic analysis was employed to elicit key concepts that were evident in the data and were viewed as essential in determining the understandings of all the participants in regards to their experience and learning outcome after attending the event. A total of 26 (26/79, 32.9%) participants answered the open item. Based on these comments, categories were coded and have been labeled as “Appreciation”, and “Training”.

### Appreciation

This theme is identified based on participant’s experience, approaches, and feedback on event process and the topics discussed. Overall, most of the participants (14/26, 53.8%) perceived the events nice and well organized. The example below demonstrates a comment from a participant.

“*Thank you for your effort and organizing such a nice event. Best Luck*”

This group represents the relative majority of comments and propositions that could be mainly grouped as follows:

1. More sessions of journal club, target more academic staff, physicians, and improve invitation strategy.
2. Improve time management by restricting the presentation and discussion section into defined period.

### Training

This category highlights the perception, comments, and suggestions of participants (12/26, 46.1%) regarding the use and training of BlackBoard (a commercial LMS system). Introduced to the Deanship of e-learning and Distance Learning in 2010 by King Saud University (KSU).^11^ BlackBoard is promoted by Medical Education department, College of Medicine to use eLearning among students, and medical educators. Most of the courses taught in the Medical College use LMS to upload related lecture notes, announcements, and deliver test grades, however, the other interactive tools, such as conferencing facilities, chat rooms, discussion boards, and evaluation tools for tests and surveys offered by LMS are still a nascent among its users at College of Medicine, King Saud University.^12^ Medical Education Journal Club conducted a session on awareness and training on BB which was attended by most of the professors, teaching and administrative staff. This session was the most successful session with highest number of attendees.

All participants of the session expressed the urge to learn and explore the LMS.

“I look forward to attend the training to use BlackBoard”

This group represents the comments and suggestions that are grouped as follows:

1. Conduct more training, and workshops on BlackBoard and other LMS.
2. Provide access to different staff members
3. Encourage teaching and administrative staff to use BlackBoard

## DISCUSSION

This study evaluated the two academic years’ experience of 1^st^ Medical Education journal club implementation at King Saud University, College of Medicine. Started in 2015, the club was joined by faculty members of medical education department and gradually took growth by welcoming the members around the college. It suggests that conducting such scholarly activity creates awareness of new trends in academics, improves knowledge, allow interactions among the staff members and promote evidence-based medical education in a medical context.

Journal club has always been a part of physician’s education since very long time.^3^ Its implementation played a vital role in development of intellectual challenges in general medicine and also in nursing, dentistry and other allied health professions.^13^,^14^ Apart from developing intellectual challenges, JC have other diverse goals, like teaching self-directed learning, keeping up-to-date in the literature, demonstrating practical clinical problems and to teach critical appraisal skills and evidence-based medicine.^1^ These learning techniques and a mix of structured and learner-directed components contributed to participants success.^8^ A study conducted by Hughes^15^ on Canadian medical students perception of clinical teaching and their desire to pursue clinical teaching skills found that students (42%) expressed confidence presenting at journal clubs along with giving presentations, bedside teaching, and teaching sensitive issues. It was observed during these 12 sessions, that the attendance rate of the event does depend on the speaker, and the topic going to discussed. It was observed that the sessions with Professor as speaker will attract the participants (39 participant) more than the event with other academic ranks. The thematic analysis was performed which welcomed the comments, and suggestions from the participants of the events. Our findings show a good level of satisfaction with the 1st MEJC in Medical Education department activity. Participants were open and were suggesting new activities and programs which shows their willingness of learning and desire for more CME activities, ideas and new tools. There are some limitations to the study: it is the experience of medical education staff only; students were not invited in the invitation list; there’s need to perform pre then post evaluation to assess learners’ self-reported changes in knowledge, skills, confidence, attitudes or behaviors where both be- fore and after information is collected at the same time. To our knowledge, MEJC of King Saud University is the first journal club in public academic centers of Saudi Arabia according to literature. This initiative will help other departments in college of medicine, and colleges outside King Saud University to gather, collaborate with national and international experts of the field to share the experience, teachings to their respective audience, staff and students.

## CONCLUSION

MEJC is an educational activity that can play important role in providing high quality healthcare teachings. We conclude that the success and consistency of MEJC depends on speaker. It commensurate the audience interest to attend and learn. In addition proper advertisement of event, and regular attendance also plays a vital role in this regard.

### Abbreviations

**JC:** Journal Club **MEJC:** Medical Education Journal Club

**KSU:** King Saud University **BB:** BlackBoard
